# 0D–1D hybrid nanoarchitectonics: tailored design of FeCo@N–C yolk–shell nanoreactors with dual sites for excellent Fenton-like catalysis†

**DOI:** 10.1039/d1sc05000a

**Published:** 2021-11-11

**Authors:** Chaohai Wang, Hongyu Wang, Jongbeom Na, Yiyuan Yao, Alowasheeir Azhar, Xin Yan, Junwen Qi, Yusuke Yamauchi, Jiansheng Li

**Affiliations:** Jiangsu Key Laboratory of Chemical Pollution Control and Resources Reuse, School of Environmental and Biological Engineering, Nanjing University of Science and Technology Nanjing 210094 People's Republic of China lijsh@njust.edu.cn; Australian Institute for Bioengineering and Nanotechnology (AIBN), The University of Queensland Brisbane Queensland 4072 Australia y.yamauchi@uq.edu.au; International Center for Materials Nanoarchitectonics (WPI-MANA), National Institute for Materials Science (NIMS) 1-1 Namiki Tsukuba Ibaraki 305-0044 Japan

## Abstract

Heterogeneous Fenton-like processes are very promising methods of treating organic pollutants through the generation of reactive oxygen containing radicals. Herein, we report novel 0D–1D hybrid nanoarchitectonics (necklace-like structures) consisting of FeCo@N–C yolk–shell nanoreactors as advanced catalysts for Fenton-like reactions. Each FeCo@N–C unit possesses a yolk–shell structure like a nanoreactor, which can accelerate the diffusion of reactive oxygen species and guard the active sites of FeCo. Furthermore, all the nanoreactors are threaded along carbon fibers, providing a highway for electron transport. FeCo@N–C nano-necklaces thereby exhibit excellent performance for pollutant removal *via* activation of peroxymonosulfate, achieving 100% bisphenol A (*k* = 0.8308 min^−1^) degradation in 10 min with good cycling stability. The experiments and density-functional theory calculations reveal that FeCo dual sites are beneficial for activation of O–O, which is crucial for enhancing Fenton-like processes.

## Abstract 2

Novel 0D–1D hybrid nanoarchitectonics consisting of FeCo@N–C yolk–shell nanoreactors are developed for Fenton-like reaction. With the multilevel advantages of this design, FeCo@N–C nano-necklaces exhibit excellent performance for BPA removal.

## Graphical abstract

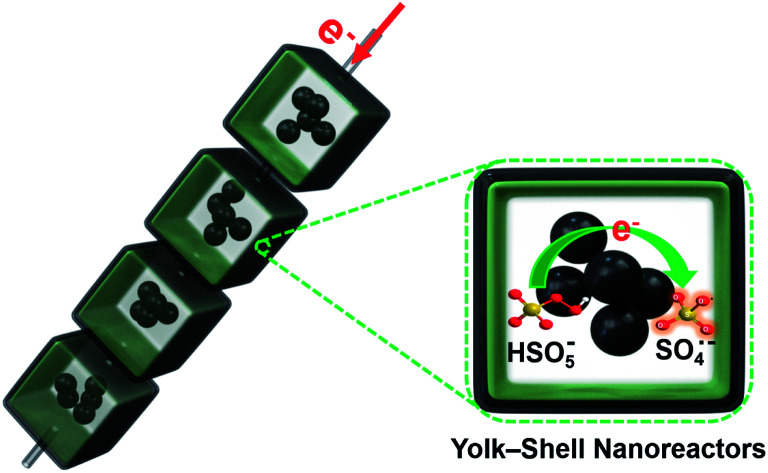

Advanced oxidation processes (AOPs) are one of the most promising strategies to eliminate organic contaminants, sustainably generating reactive oxygen species (ROS) to ideally destroy all non-biodegradable, recalcitrant, toxic, or membrane-permeable organic impurities.^1–4^ Among these AOPs, sulfate radical (SO_4_˙^−^)-based Fenton-like processes have gained increasing attention as a water treatment strategy because of the strong oxidation potential of SO_4_˙^−^ (3.1 V *vs.* normal hydrogen electrode) at wider pH ranges. SO_4_˙^−^ is mainly produced by physical or chemical methods for activation of persulfate salts, such as peroxymonosulfate (PMS) and persulfate.^5–9^ Over the past two decades, heterogeneous catalysis has emerged as the most effective approach to water treatment, with much effort dedicated to developing better catalysts, including transition metal-based and carbonaceous materials.^10,11^ Unfortunately, most metal-based catalysts suffer from leaching of toxic metal ions, which can thwart their practical application,^12,13^ and although carbonaceous catalysts produce no secondary pollution, their cycle performance is always depressed.^14^ There is therefore an urgent need to find robust catalysts with adequate activity and stability for Fenton-like processes.

To achieve superior performance, an ideal Fenton-like catalyst should contain oxidants with favorably reactive centers for cleavage of peroxyl bonds (O–O), have structure optimized for target pollutant attraction, and have chainmail to protect the vulnerable active sites for long periods.^15–17^ Recent studies have demonstrated Co–N–C active sites prefer to activate the O–O of PMS.^18^ Furthermore, introducing Fe-doping into the Co–N–C system not only suppresses Co^2+^ leaching, but also modulates the pyrrolic-*N* content, which is the adsorption site for capture of bisphenol A (BPA).^19^ We previously discovered that Co@C yolk–shell nanoreactors could enhance the catalytic activity because of the confinement effect in the nano-spaces between the core and shell, while the carbon shell acted like a chainmail protecting the Co active sites, keeping them highly reactive after five cycles.^20,21^

Combining different kinds of materials to generate novel hybrid material interfaces can enable the creation of new kinds of chemical and physical functionalities that do not currently exist. However, one cannot simply mix these materials in an uncontrolled manner, because the ensemble of interfaces created by random mixing tends to favour thermodynamically stable interfaces that are functionally less active. Therefore, to prepare new materials with high functionality, it is necessary to carefully control the hybridization of components in interfacial regions with nanometric or atomic precision. By further hybridization of different components *e.g.*, zero to one dimension (0D–1D) hybrid structures, we can prepare the structure to increase not only the specific surface area but also the interfacial region between different materials.

In this work, we report novel 0D–1D hybrid nanoarchitectonics (necklace-like structures) consisting of FeCo@N–C yolk–shell nanoreactors as a PMS activator for Fenton-like processes. This catalyst has multilevel advantages: (i) each FeCo@N–C unit is a well-formed yolk–shell nanoreactor, which can guarantee sufficient contact of reactants and active sites, as well as defend them for good durability; (ii) all single nanoreactors are threaded along the carbon fibers, providing a highway for electron transport; and (iii) all the carbon fibers constructed into a thin film with macroscopic structure, which overcomes the complex recyclability of powder catalysts. Benefiting from favorable composition and unique structure, the FeCo@N–C catalyst delivers excellent performance for BPA removal *via* activation of PMS accompanied with good stability.

The synthesis processes of necklace-like nanoarchitecture containing FeCo@N–C yolk–shell nanoreactors are illustrated in Fig. 1a. First, uniform Fe–Co Prussian blue analogue (Fe–Co PBA) nanocubes with an average size of 800–900 nm (Fig. 1b) are encapsulated in polyacrylonitrile (PAN) nanofibers by electrospinning. The obtained necklace-like FeCo PBA–PAN fibers (Fig. 1c) are then pyrolyzed at 800 °C in N_2_ atmosphere to produce FeCo@N–C nano-necklaces. The scanning electron microscopy (SEM) image (Fig. 1d) of the FeCo@N–C shows this necklace-like morphology with its large aspect ratio, with the FeCo@N–C particles strung along the PAN-derived carbon fibers. A broken particle (Fig. 1e) shows that the FeCo@N–C has a yolk–shell architecture, which is also identified by transmission electron microscopy (TEM). Fig. 1f and g show the well-defined space between the inner yolk and outer shell, which is attributed to the volume shrinkage of the original Fe–Co PBAs. During pyrolysis, Fe–Co PBA is reduced to FeCo (inner yolk) and PAN is carbonized (outer carbon shell), resulting in the unique necklace-like nanoarchitecture.^22–24^ The high-resolution TEM in Fig. 1h shows a lattice fringe of 0.20 nm, which matches well with the (110) plane of FeCo alloy.^25^ The scanning transmission electron microscopy (STEM) image (Fig. 1i) and corresponding elemental map (Fig. 1j) indicate that FeCo nanocrystals are well distributed in the inner core with some small FeCo nanocrystals located on external carbon shells. Furthermore, the control samples of Fe@N–C and Co@N–C nano-necklaces, prepared by only replacing the Fe–Co PBA nanocubes with Fe–Fe PB and Co–Co PBA (Fig. S1†), also demonstrate the versatility of this synthetic strategy. The formation of hierarchical porous structure, beneficial to the PMS transportation on the surface of catalysts, could be determined by N_2_ adsorption–desorption isotherms and corresponding pore volume analysis (Fig. S2 and Table S1†).

**Fig. 1:**
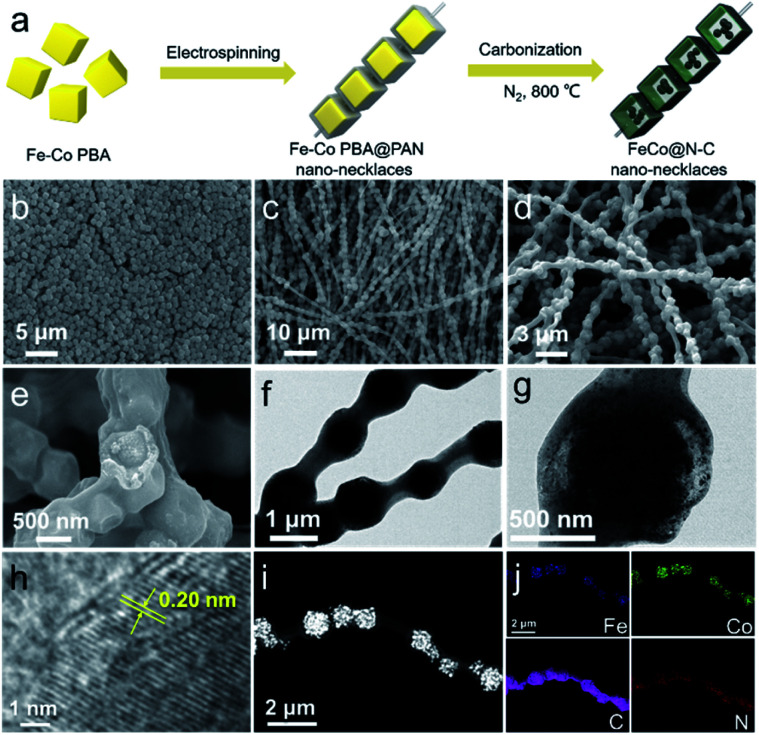
(a) Preparation of FeCo@N–C necklace-like nanoarchitecture. SEM images of (b) Fe–Co PBA cubic particles and (c) the electrospun FeCo PBA–PAN fibers. (d and e) SEM, (f and g) TEM, and (h) high-resolution TEM images of FeCo@N–C nano-necklaces. (i) STEM and (j) the corresponding elemental mappings of C, N, Fe, and Co.

The X-ray diffraction patterns of the as-prepared products are depicted in Fig. S3,† with one prominent diffraction peak centered at 44.8° corresponding to the (110) lattice plane of FeCo alloy. All the products also have a characteristic signal at 26°, implying that graphite carbon is formed during pyrolysis. Raman spectroscopy further analyzed the crystal structures and defects of the FeCo@N–C nano-necklaces (Fig. S4†), where peaks found at 1349 cm^−1^ and 1585 cm^−1^ index the disordered (D band) and graphitic carbon (G band), respectively.^26^ X-ray photoelectron spectroscopy investigated the composition and valence band spectra of FeCo@N–C nano-necklaces. The survey spectrum (Fig. S5a†) reveals the presence of Fe (1.4%), Co (1.2%), C (86.4%), N (4.5%), and O (6.5%) in the composite. The high-resolution N 1s spectrum (Fig. S5b†) exhibits broad peaks at 398.1, 401.1, and 407.4 eV, corresponding to the pyridinic-*N*, graphitic-*N*, and σ* excitation of C–N, respectively.^27^ The high-resolution Fe 2p spectrum (Fig. S5c†) shows a broad peak at 707.4 eV, attributed to Fe^0^. Similarly, the 777.5 eV peak observed in the Co 2p spectrum (Fig. S5d†) corresponds to Co^0^, implying that FeCo dual sites have formed.^28^ The oxidation state of these sites was investigated by ^57^Fe Mössbauer spectroscopy, which found a sextet in the Mössbauer spectrum of the FeCo@N–C nano-necklaces attributed to FeCo dual sites (Fig. 2a and Table S2†).^29^ The coordination environment of the FeCo dual sites was also verified by X-ray absorption fine structure (XAFS) spectroscopy. Fig. 2b shows that the X-ray absorption near-edge structure (XANES) spectra of the Fe K-edge, which demonstrates a similar near-edge structure to that of Fe foil, illustrating that the main valence state of Fe in FeCo@N–C nano-necklaces is Fe^0^. Furthermore, the extended-XAFS (EXAFS) spectra (Fig. 2c) displays a peak at 1.7 Å, which is ascribed to the Fe–N bond, and a remarkable peak at approximately 2.25 Å corresponding to the metal–metal band.^10,30^ The Co K-edge and EXAFS spectra (Fig. S6†) also confirm the presence of Co–N and the metal–metal band. These results provide a potential structure of the FeCo dual sites in the FeCo@N–C nano-necklaces, as illustrated in Fig. 2d.

**Fig. 2:**
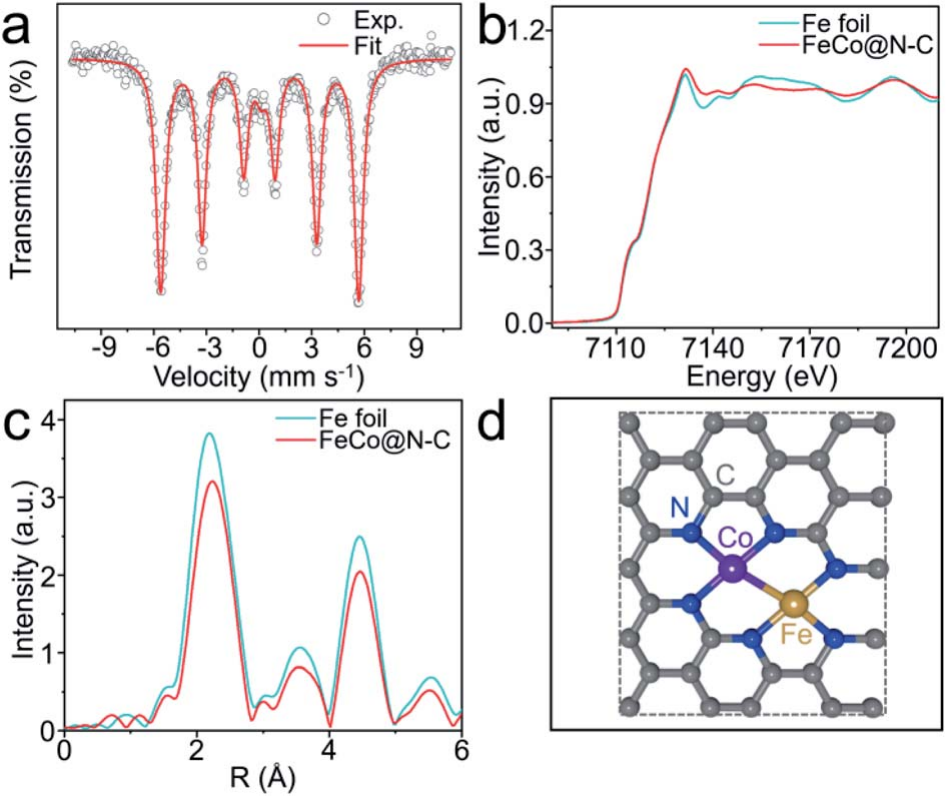
(a) ^57^Fe Mössbauer spectra of FeCo@N–C nano-necklaces at 298 K. (b) Fe K-edge XANES spectra of FeCo@N–C nano-necklaces and Fe foil. (c) Corresponding Fourier transformed *k*^3^-weighted of the EXAFS spectra for Fe K-edge. (d) Possible structure of the FeCo dual sites.

This dual-metal center and necklace-like structure may be beneficial to enhance catalytic performance. Fig. 3a shows the Fenton-like performance for BPA degradation compared to Fe@N–C nano-necklaces, Co@N–C nano-necklaces, and FeCo@N–C particles (Fe–Co PBA directly carbonized without electrospinning). Here, the FeCo@N–C nano-necklaces display a higher catalytic performance, with BPA completely removed in 7 min. To clearly compare their catalytic behavior, the kinetics of BPA degradation was fitted by the first-order reaction. As shown in Fig. 3b, FeCo@N–C nano-necklaces exhibit the highest apparent rate constant (*k* = 0.83 min^−1^), which is approximately 6.4, 2.6, and 1.2 times that of FeCo@N–C particles, Fe@N–C nano-necklaces, and Co@N–C nano-necklaces, respectively. The significantly enhanced performance of FeCo@N–C nano-necklaces suggests that the FeCo dual sites and necklace-like nanoarchitecture are crucial. Furthermore, the concentration of BPA and PMS in the solution is higher than that in yolk–shell nanoreactor, resulting a concentration gradient which helps to accelerate the diffusion rates of reactants (Fig. 3c).^31,32^ For these nano-necklaces, the carbon shell acts like a chainmail protecting the FeCo active sites from attack by molecules and ions, and all the nanoreactors are threaded along the carbon fibers, providing a highway for electron transport, which is important for SO_4_˙^−^ generation (SO_4_˙^−^ production as eqn, HSO_5_^−^ + e^−^ → SO_4_˙^−^ + OH^−^). Electrochemical impedance spectroscopy further confirms the good conductivity of the FeCo@N–C nano-necklaces (Fig. 3d). In addition, the concentration of metal-ion leaching and cycling performance (Fig. 3e and f) reveal the high reusability of FeCo@N–C nano-necklaces, with 95% BPA removal in 20 min after five cycles, which is also proved by the SEM and TEM characterization (Fig. S7†). The effect of other reaction parameters on the BPA degradation, such as pH, reaction temperature, PMS or catalysts dosage, and common anions, were investigated in detail (Fig. S8–S11†). All the results demonstrate that FeCo@N–C nano-necklaces deliver a better performance for PMS catalysis. In addition, the turnover frequency (TOF) value of FeCo@N–C nano-necklaces is 5.5 min^−1^ for BPA degradation, which is higher than many previously reported catalysts (detailed catalytic performance comparison as shown in Table S3†).

**Fig. 3:**
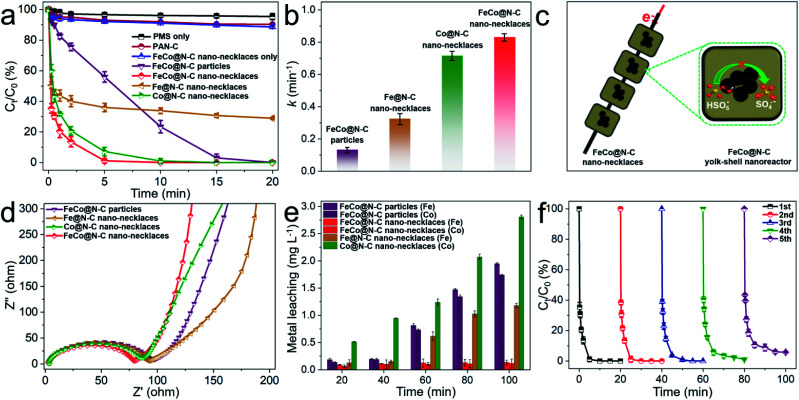
(a) BPA degradation efficiency in different reaction systems and (b) the corresponding reaction rate constants. (c) Schematic illustration of PMS activation in FeCo@N–C nano-necklaces. (d) Nyquist plots of the catalysts. (e) The metal leaching in different reaction systems. (f) Cycling performance of FeCo@N–C nano-necklaces for BPA removal. Reaction conditions: [catalyst] = 0.15 g L^−1^, [BPA] = 20 mg L^−1^, [PMS] = 0.5 g L^−1^, *T* = 298 K, and initial pH = 7.0.

To examine the enhanced catalytic activity, radical quenching experiments were conducted. As shown in Fig. 4a, when NaN_3_ is added to the reaction solution as a scavenger for ^1^O_2_, there is no significant reduction of BPA decomposition, implying that non-radicals are not the dominant reactive species. By comparison, when *tert*-butanol (TBA) (radical scavenger for ˙OH) is added, there is a slight (2.8%) decrease in BPA removal. However, if methanol (radical scavenger for SO_4_˙^−^ and ˙OH) is added, the efficiency of BPA degradation declines by up to 59.2%, indicating that the major radicals generated from the PMS activation are SO_4_˙^−^;^33^ the presence of these radicals is also verified by electron paramagnetic resonance (EPR) (Fig. 4b). Furthermore, the significant inhibition ratio can be observed when KI (quencher for the surface) is added, demonstrating that BPA degradation is mainly attributed to reactions with SO_4_˙^−^, which is produced by a surface catalytic process.^34^

**Fig. 4:**
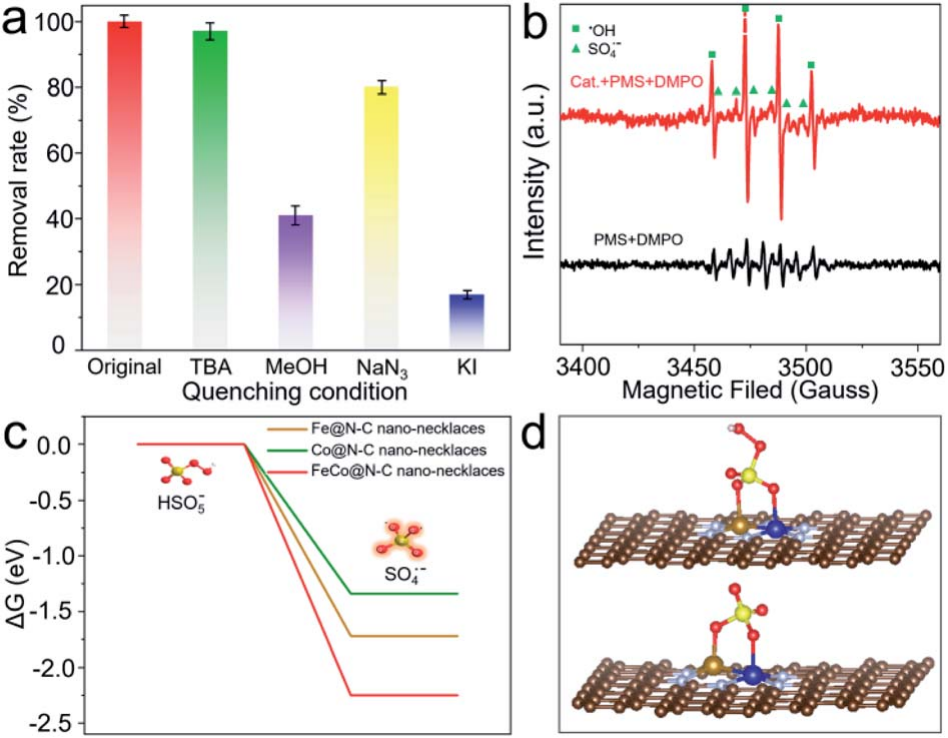
(a) Effects of the radical scavengers on BPA degradation. (b) EPR spectra of SO_4_˙^−^ and ˙OH. (c) The energy profiles of PMS on FeCo@N–C nano-necklaces surface. (d) Optimized configurations of PMS adsorbed on FeCo@N–C nano-necklaces.

Density-functional theory was applied to calculate the surface energy of PMS activation at FeCo dual sites (Fig. 4c, d and S12†). The dissociation barrier of PMS into SO_4_˙^−^ and OH^−^ is −2.25 eV, which is much lower than that on an Fe or Co single site, suggesting that cleavage of O–O bonds of PMS occurs more easily on FeCo dual sites. This is because FeCo dual sites provide two anchoring sites for the dissociated O atoms, leading to more efficient activation of O–O. The FeCo@N–C nano-necklaces can reduce the energy barrier of O–O bond breaking, which results in high activity for PMS activation and thus high productivity of SO_4_˙^−^.

## Conclusions

We have demonstrated novel FeCo@N–C nano-necklaces as catalysts for Fenton-like processes, with their FeCo dual sites enhancing the cleavage O–O bond of PMS. Their well-defined yolk–shell nanoreactors based on necklace-like architectures also exhibit superior cycling stability. Our findings not only develop a new catalyst with high performance for Fenton-like processes, but also propose an explanation for the improved catalytic performance provided by dual metal sites.

## Author contributions

C. W. synthesized the samples and wrote this manuscript. H. W., Y. Y., and X. Y. characterized the materials. J. N., A. A., and J. Q. revised the manuscript. J. L. and Y. Y. conceived of the experiments.

## Conflicts of interest

The authors declare no competing financial interest.

## Supplementary Material

SC-012-D1SC05000A-s001
