# Supplementary material for: 0D–1D hybrid nanoarchitectonics: tailored design of FeCo@N–C yolk–shell nanoreactors with dual sites for excellent Fenton-like catalysis
Source: Chem Sci. 2021 Nov 11;12(46):15418–22. doi: 10.1039/d1sc05000a (PMC8635224; doi:10.1039/d1sc05000a)
Supplement: SC-012-D1SC05000A-s001 [file SC-012-D1SC05000A-s001.pdf]

Electronic Supplementary Material (ESI) for Chemical Science.
This journal is © The Royal Society of Chemistry 2021

Electronic Supplementary Information

0D-1D hybrid nanoarchitectonics: Tailored design of FeCo@N-C yolk–shell nanoreactors with dual sites for excellent Fenton-Like catalysis

Chaohai Wang,[ab] Hongyu Wang,[a] Jongbeom Na,[b] Yiyuan Yao,[a] Alowasheeir Azhar,[c] Xin Yan,[a] Junwen Qi,[a] Yusuke Yamauchi*,[bc] Jiansheng Li*[a]

[a] Jiangsu Key Laboratory of Chemical Pollution Control and Resources Reuse, School of Environmental and Biological Engineering, Nanjing University of Science and Technology, Nanjing 210094, People's Republic of China. E-mails: lijsh@njust.edu.cn

[b] Australian Institute for Bioengineering and Nanotechnology (AIBN) and School of Chemical Engineering, The University of Queensland, Brisbane, QLD 4072, Australia

E-mails: y.yamauchi@uq.edu.au

[c] International Center for Materials Nanoarchitectonics (WPI-MANA), National Institute for Materials Science (NIMS), 1-1 Namiki, Tsukuba, Ibaraki 305-0044, Japan

Table of contents

1. Material Synthesis and Characterization

2. Catalytic activity measurements

3. Supporting Fig.s and Tables

4. References

1. Material Synthesis and Characterization

Chemicals

Cobalt acetate ($Co(CH_3COO)_2{\cdot}4H_2O$), potassium hexacyanoferrate trihydrate ($K_4[Fe(CN)_6]{\cdot}3H_2O$), potassium hexacyanocobaltate(III) ($K_3[Co(CN)_6]$), and Polyvinyl pyrrolidone (PVP, K30) were purchased from Sinopharm Chemical Reagent Co., Ltd. (Shanghai, China). Potassium peroxymonosulfate (PMS) and polyacrylonitrile (PAN, MW = 150000) were purchased from Sigma-Aldrich. Ferrous sulfate heptahydrate ($FeSO_4{\cdot}7H_2O$), tertbutyl alcohol (TBA), *N,N*-dimethylformamide (DMF), and other organic solvents were acquired from Nanjing Chemical Reagent Co., Ltd. (Nanjing, China).

Experimental

Synthesis of PB and PBA Nanoparticles

Fe-Fe PB, Fe-Co PBA, and Co-Co PBA nanoparticles were prepared according to the previous method with a little modification (*Chem. Commun.* 2015, 51, 10479-10482). Typically, 15.2 g PVP was added into 200 mL of DI water with continuous stirring. When PVP was completely dissolved, 1.8 mL HCl was dropped into PVP solution and stirred for 10 min. Then, 0.44 g $K_4[Fe(CN)_6]{\cdot}3H_2O$ was added into the mixture and stirred for another 1 h at room temperature. After that, the above solution was transferred into a 250 mL Teflon-lined stainless autoclave, heated to 80°C for 24 h to produce Prussian blue (Fe-Fe PB).

For preparation of Prussian blue analogues (PBAs), 0.332 g $K_3[Co(CN)_6]{\cdot}3H_2O$ and 6.0 g PVP were dissolved in 200 mL of DI water. Then a peristaltic pump was used to drop 200 mL of 2.1 $mg{\cdot}mL^{-1}$ $FeSO_4{\cdot}7H_2O$ or 1.87 $mg{\cdot}mL^{-1}$ $Co(CH_3COO)_2{\cdot}4H_2O$ solution in the above solution with a flow rate of 4 $mL{\cdot}min^{-1}$. Fe-Co PBA or Co-Co PBA was obtained after stirring 24 h under room temperature. The products were collected by centrifugation and washed 3 times by DI water and anhydrous ethanol, and finally dried up at 60°C.

Synthesis of PBA-PAN fibers

1.3 g PBA nanoparticles were added into 5.0 mL DMF with sonication until it was well dispersed. Then, 0.65 g PAN was added into the solution with stirring at 60 °C for 4 h to obtain the electrospinning precursor. The electrospinning process was carried out by applying a high positive voltage (10 kV) with a collecting distance 15 cm. The injection speed was fixed at 0.15 mm min^{-1}.

Synthesis of Fe@N-C, Co@N-C, and FeCo@N-C nano-necklaces

The above obtained PBA-PAN fibers were placed in a tube furnace and then heated to the 800 °C for 4 h at the heating rate of 5 °C min^{-1} in N_2 atmosphere.

Synthesis of FeCo@N-C particles and PAN-C

The control sample FeCo@N-C particles were prepared from directly carbonized Fe-Co PBA. PAN fibers were prepared without adding PBA particles in the precursor solution, following with same and pyrolysis as above to get PAN-C.

Characterization

The morphology of the samples was investigated by TEM (FEI T20), SEM (FEI 250), STEM (Tecnai G2 F30 S-TWIN). The XPS (X-ray photoelectron spectroscopy) spectra were obtained by using a PHI Quantera II ESCA System with Al Kα radiation at 1486.8V. N_2 adsorption and desorption isotherms were measured using Micromeritics ASAP-2020 at liquid nitrogen temperature (77 K). The composition was examined by XRD instrument (BRUKER D8, Cu Kα) at 40 kV and 40 mA (λ=1.5418 Å). XAFS spectra at the Co K-edge (7709 eV) and Fe K-edge (7112 eV) were measured at the beamline 1W1B station of the Shanghai Synchrotron Radiation Facility, China. The Co K-edge XANES data were recorded in a fluorescence mode. Co foil was used as references. The Fe K-edge XANES data were recorded in a fluorescence mode. Fe foil was used as references. The extended X-ray absorption fine structure (EXAFS) data were calculated utilizing FEFF 9.0. The Mössbauer measurements were performed at room temperature using a conventional spectrometer (Germany, Wissel MS-500) in transmission geometry with constant acceleration mode. A ^{57}Co (Rh) source with activity of 25 mCi was used. The velocity calibration was done with a room temperature α-Fe absorber. The spectra were fitted by the software Recoil using Voigt-based Fitting (VBF) analysis.

Computational Framework

Spin-polarized DFT calculations were performed with the Vienna ab initio simulation package (VASP). The generalized gradient approximation proposed by Perdew-Burke-Ernzerhof is selected for the exchange-correlation potential. The cut-off energy for plane wave is set to 400 eV. The energy criterion is set to 10^{-5} eV in iterative solution of the Kohn-Sham equation. The Brillouin zone integration is performed at the Gamma point. All the structures are relaxed until the residual forces on the atoms have declined to less than 0.05 eV/Å. The Gibbs free energies

are defined as

$$\Delta G=E+E_{ZPE}-TS,$$

where E, E_{ZPE}, and TS refer to the total energy of the system, the zero-point energy, and the entropy.

2. Catalytic activity measurements

50 mg PMS was first added to 100 mL 20 mg L^{-1} bisphenol A (BPA) solution with stirring for 5 min to ensure that the PMS dissolved completely. 15 mg catalysts were added to the above solution. 1.5 mL of solution was carried out and quenched with 1.5 mL methanol. The remaining BPA were tested by high-performance liquid chromatograph (HPLC) (Waters, e2695). All control experiments were performed under the same conditions. The EPR (Bruker EMX 10/12) experiments were performed to record signals of the radicals using DMPO and $^1O_2$ using TMP as a spin-trapping agent. The leaching of metal ions was analyzed by an inductively coupled plasma-optical emission spectrometer (ICP-OES) (Optima 7000DV, Perkin Elmer). The electrochemistry impedance (EIS) measurements were carried out in 5 mV ac amplitude (CHI660E, Shanghai, China). The turnover frequency (TOF) was calculated through dividing the reaction rate of pollutant degradation by the catalyst concentration.

3. Supporting Figures and Tables

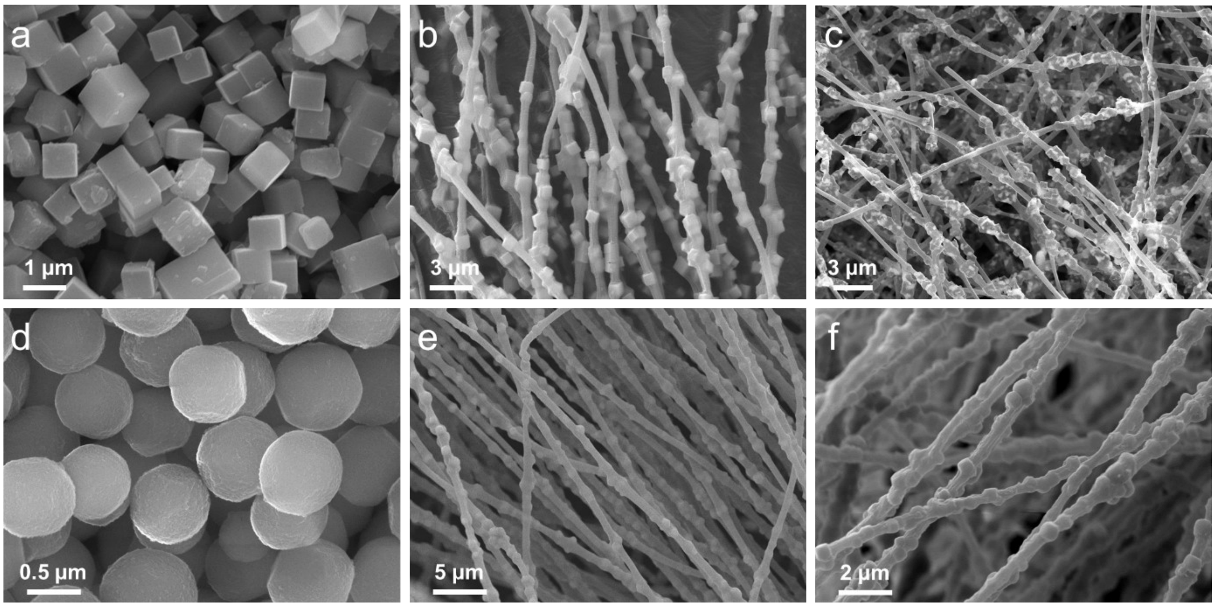

Fig. S1 SEM images of (a) Fe-Fe PB particles, (b) the electrospun FeFe PBA-PAN fibers, and (c) Fe@N-C nano-necklaces. SEM images of (d) Co-Co PBA particles, (e) the electrospun CoCo PBA-PAN fibers, and (f) Co@N-C nano-necklaces.

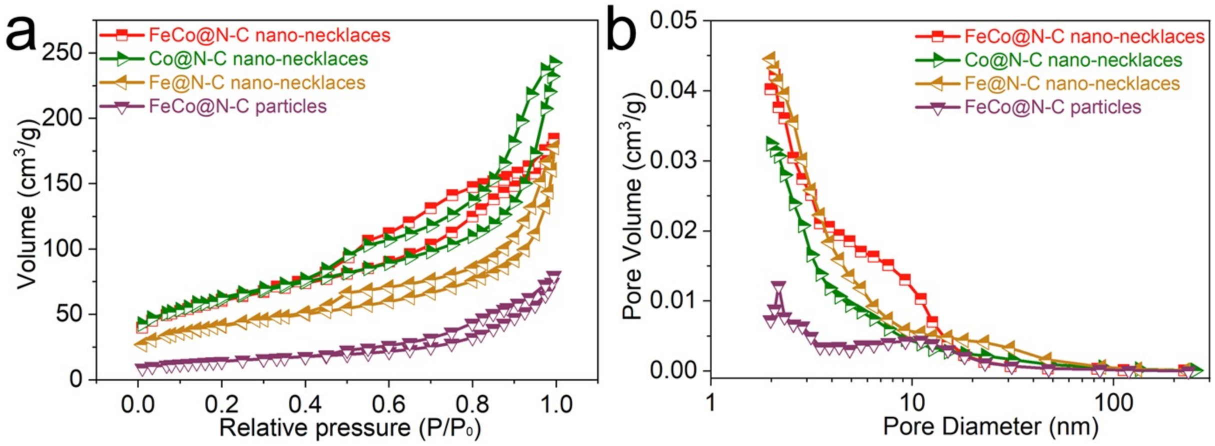

Fig. S2 (a) N_2 adsorption-desorption isotherms and (b) pore size distributions of FeCo@N-C particles, Fe@N-C nano-necklaces, Co@N-C nano-necklaces, and FeCo@N-C nano-necklaces.

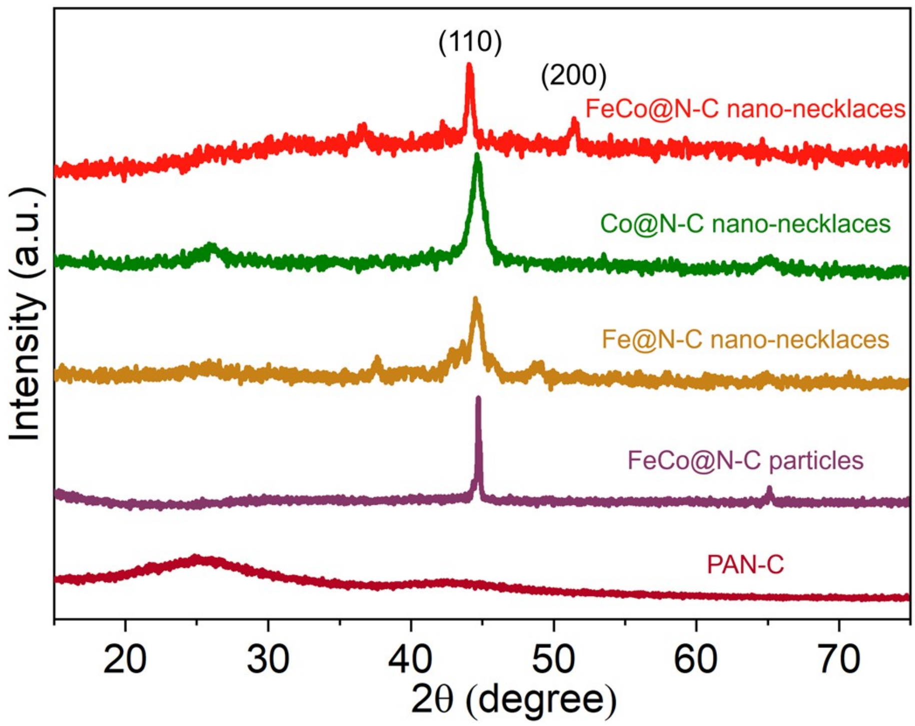

Fig. S3 XRD patterns of PAN-C, FeCo@N-C particles, Fe@N-C nano-necklaces, Co@N-C nano-necklaces, and FeCo@N-C nano-necklaces.

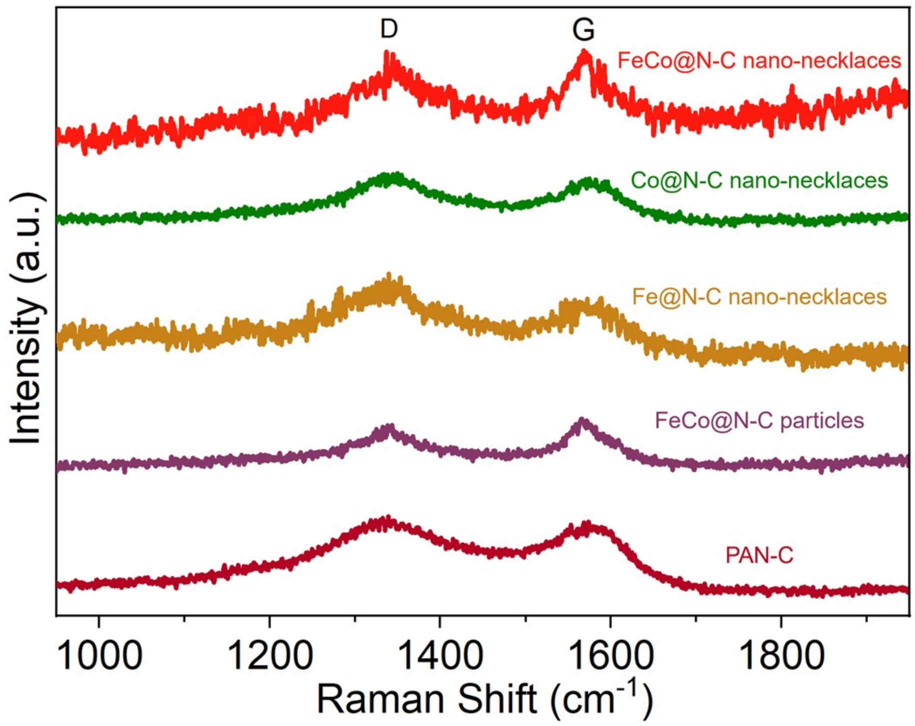

Fig. S4 Raman spectra of PAN-C, FeCo@N-C particles, Fe@N-C nano-necklaces, Co@N-C nano-necklaces, and FeCo@N-C nano-necklaces.

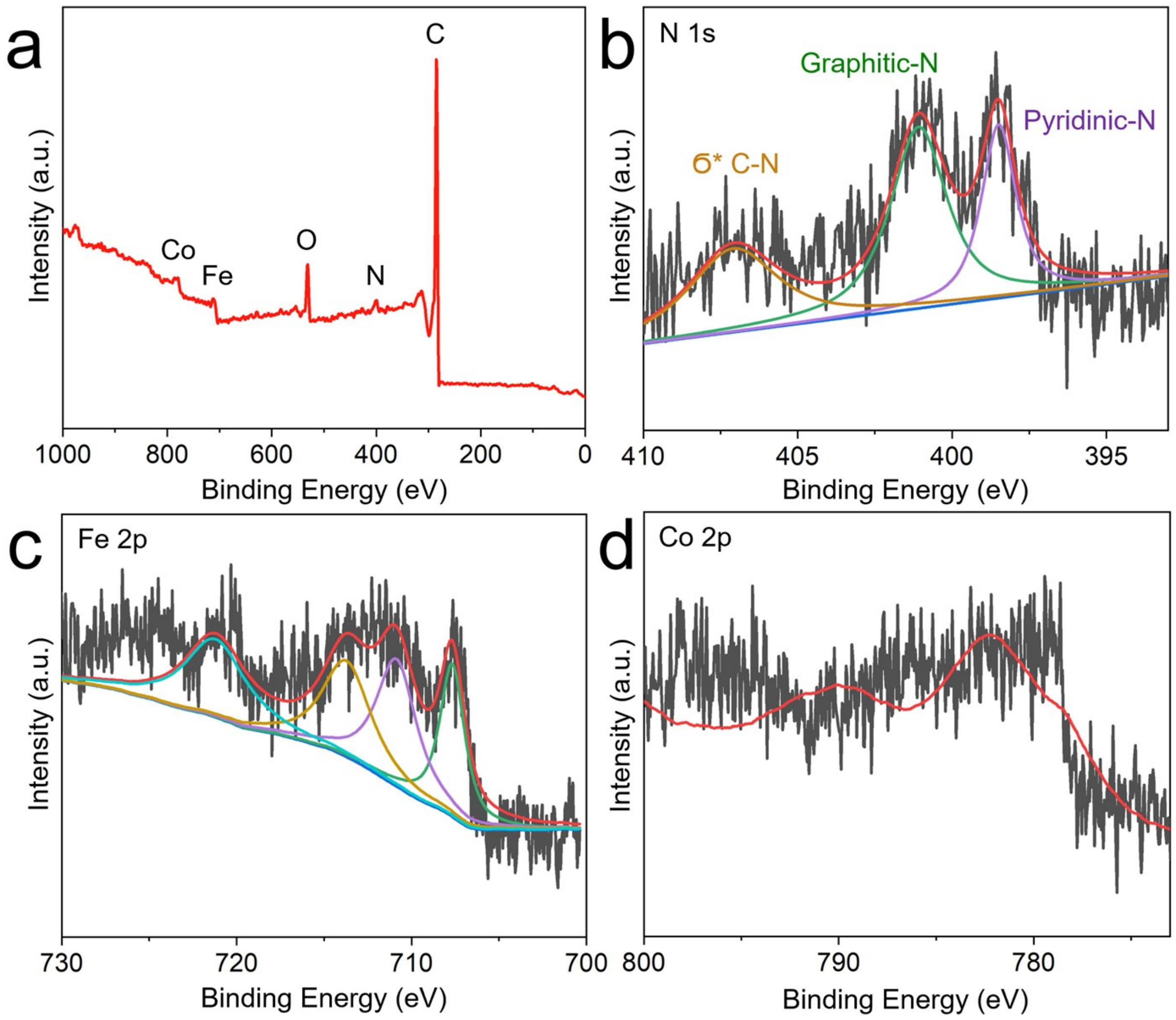

Fig. S5 XPS spectra for the (a) survey scan, b) N 1s, (c) Fe 2p, and (d) Co 2p regions of FeCo@N-C nano-necklaces.

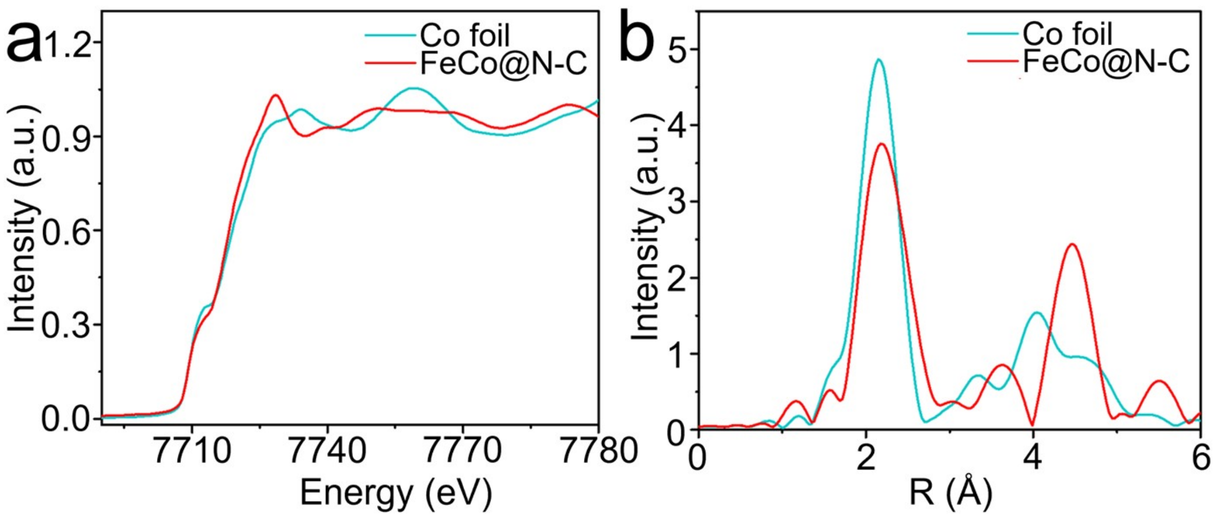

Fig. S6 Co K-edge XANES spectra of FeCo@N-C nano-necklaces and Co foil. (c) Corresponding Fourier transformed k^3-weighted of the EXAFS spectra for Co K-edge.

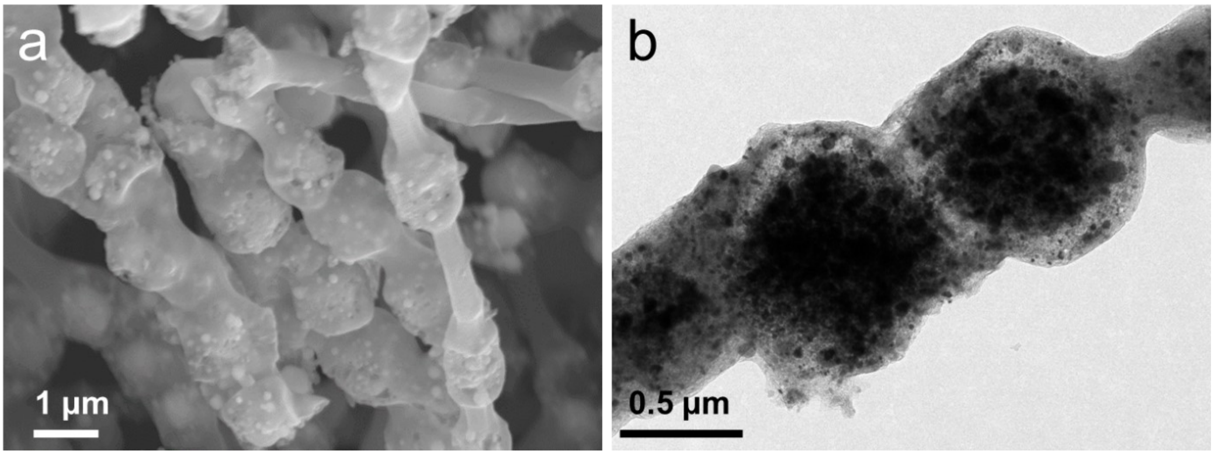

Fig. S7 (a) SEM image and (b) TEM image of FeCo@N-C nano-necklaces after 5 cycles test.

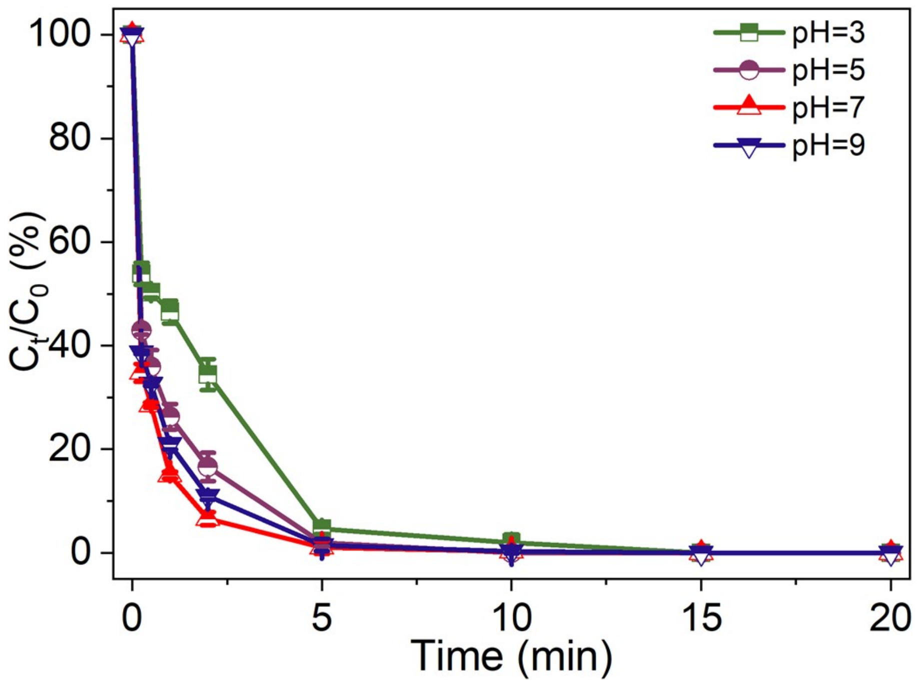

Fig. S8 Effects of initial pH ([FeCo@N-C nano-necklaces] = 0.15 g L^{-1}, [BPA] = 20 mg L^{-1}, [PMS] = 0.5 g L^{-1}, T = 298 K).

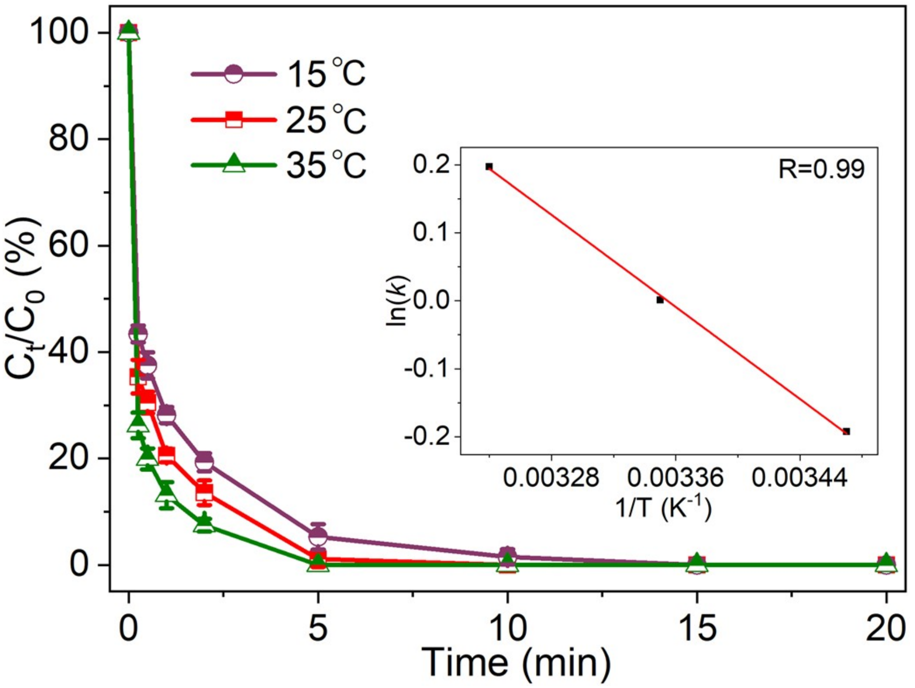

Fig. S9 Effects of temperature ([FeCo@N-C nano-necklaces] = 0.15 g L^{-1}, [BPA] = 20 mg L^{-1}, [PMS] = 0.5 g L^{-1}, pH = 7.0), the inset showing the Arrhenius plot.

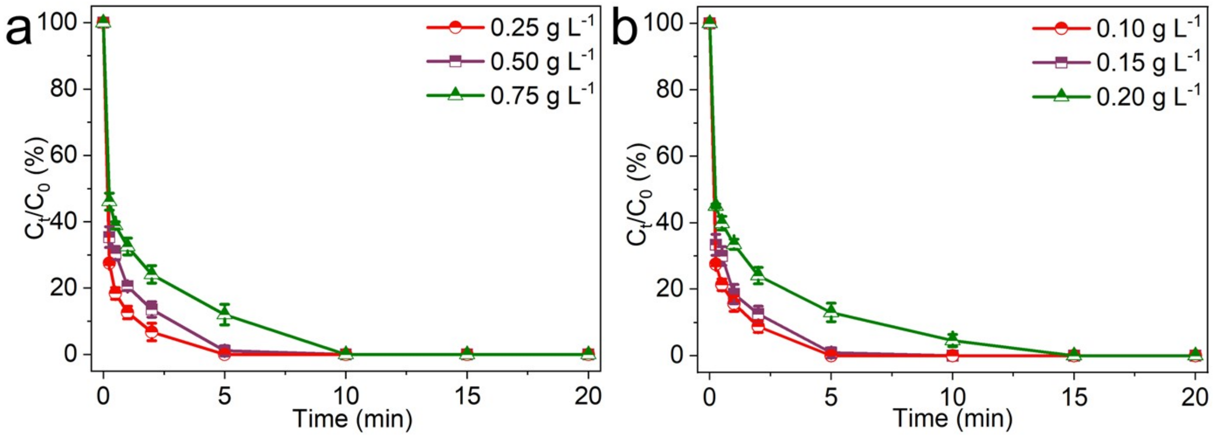

Fig. S10 Effects of (a) PMS dosage ([FeCo@N-C nano-necklaces] = 0.15 g L^{-1}, [BPA] = 20 mg L^{-1}, pH = 7.0); (b) Catalyst loading ([BPA] = 20 mg L^{-1}, [PMS] = 0.5 g L^{-1}, pH = 7.0).

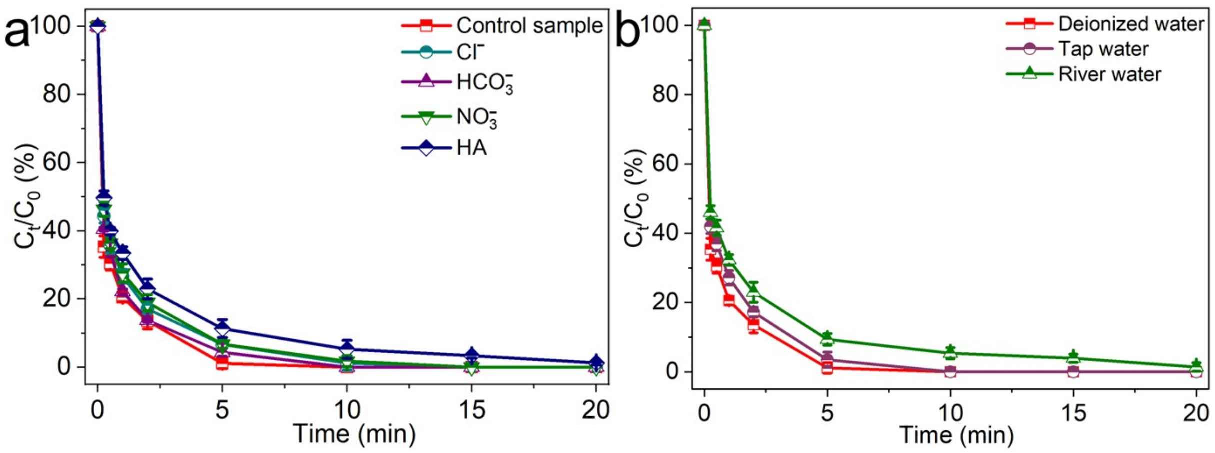

Fig. S11 Effects of Cl^-, HCO_3^-, NO_3^-, and HA on BPA removal. (b) BPA removal in the actual water system ([FeCo@N-C nano-necklaces] = 0.15 g L^{-1}, [BPA] = 20 mg L^{-1}, T = 298 K, pH = 7.0).

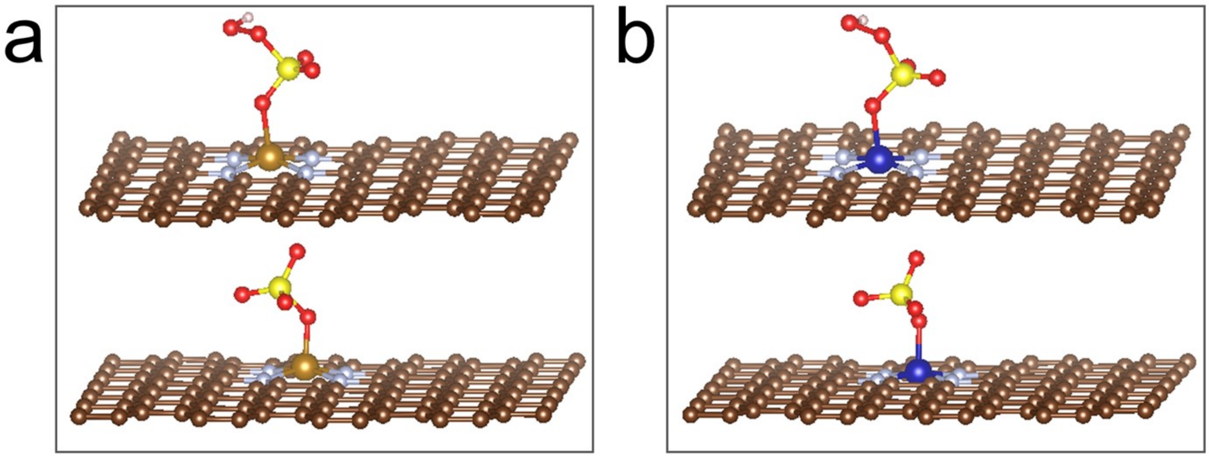

Fig. S12 Optimized configurations of PMS adsorbed on (a) Fe@N-C nano-necklaces and (b) Co@N-C nano-necklaces.

Table S1. The BET surface area and pore volume of as-prepared catalysts.

Catalysts	S_{BET} (m^2 g^{-1})	Pore volume (cm^3 g^{-1})
Fe@N-C nano-necklaces	217.1	0.32
Co@N-C nano-necklaces	146.6	0.20
FeCo@N-C nano-necklaces	214.7	0.27
FeCo@N-C particles	50.8	0.12

Table S2. Mössbauer parameters of FeCo@N-C nano-necklaces.

Sample	δ	$B_{hf,m}$	ΔE_Q
FeCo@N-C nano-necklaces	0.028	349.84	0.009

δ: isomer shift, $B_{hf,m}$: hyperfine field, ΔE_Q: quadrupole splitting.

Table S3. The catalytic performance comparison of recently reported catalysts for BPA removal.

Catalyst ($g\ L^{-1}$)	PMS ($g\ L^{-1}$)	BPA ($m\ L^{-1}$)	Removal efficiency	References
FeCo@N-C nano-necklaces (0.1)	0.5	20	100% (6 min)	This work
Co@N-C nano-necklaces (0.1)	0.5	20	100% (8 min)	This work
Fe@N-C nano-necklaces (0.1)	0.5	20	80% (20 min)	This work
Co-SAs (0.2)	0.4	20	81.6% (12 min)	1
HCCNs (0.1)	0.15	20	98% (within 6 min)	2
MnFeO (0.1)	0.2	10	95% (within 30 min)	3
FeCo-NC-2 (0.1)	0.2	20	100% (4 min)	4
O-CN (1.0)	6.2	11.2	100% (45 min)	5
$Fe_1Mn_5Co_4$-N@C (0.1)	0.2	20	100% (10 min)	6
SA-Fe-NC (0.05)	1.2	22.4	100% (3 min)	7
NGC700 (0.1)	0.2	20	98% (4 min)	8
Fe-MMT (2.5)	0.6	1.1	100% (60 min)	9
Fe-doped g-C_3N_4 (0.1)	0.6	10	100% (4 min)	10
Co@N-C-KNO_3 (0.05)	0.6	50	100% (10 min)	11
B-OMC (0.2)	0.6	20	91% (60 min)	12
NGA (0.05)	2	10	100% (30 min)	3
SiM-1 (0.4)	0.1	10	99% (30 min)	14
NPSC-700 (0.06)	0.4	25	90.1% (30 min)	15
ce-MoS_2 (0.05)	0.5	2	100% (20 min)	16

4. References

1. Gao, Y.; Yang, C.; Zhou, M.; He, C.; Cao, S.; Long, Y.; Li, S.; Lin, Y.; Zhu, P.; Cheng, C., Transition Metal and Metal-Nx Codoped MOF-Derived Fenton-Like Catalysts: A Comparative Study on Single Atoms and Nanoparticles. *Small* **2020**, *16* (50), 2005060.

2. Zhang, M.; Xiao, C.; Yan, X.; Chen, S.; Wang, C.; Luo, R.; Qi, J.; Sun, X.; Wang, L.; Li, J., Efficient Removal of Organic Pollutants by Metal-organic Framework Derived Co/C Yolk-Shell Nanoreactors: Size-Exclusion and Confinement Effect. *Environ. Sci. Technol.* **2020**, *54* (16), 10289-10300.

3. Huang, G. X.; Wang, C. Y.; Yang, C. W.; Guo, P. C.; Yu, H. Q., Degradation of Bisphenol A by Peroxymonosulfate Catalytically Activated with $Mn_{1.8}Fe_{1.2}O_4$ Nanospheres: Synergism between Mn and Fe. *Environ. Sci. Technol.* **2017**, *51* (21), 12611-12618.

4. Li, X.; Huang, X.; Xi, S.; Miao, S.; Ding, J.; Cai, W.; Liu, S.; Yang, X.; Yang, H.; Gao, J.; Wang, J.; Huang, Y.; Zhang, T.; Liu, B., Single Cobalt Atoms Anchored on Porous N-Doped Graphene with Dual Reaction Sites for Efficient Fenton-like Catalysis. *J. Am. Chem. Soc.* **2018**, *140* (39), 12469-12475.

5. Gao, Y.; Zhu, Y.; Lyu, L.; Zeng, Q.; Xing, X.; Hu, C., Electronic Structure Modulation of Graphitic Carbon Nitride by Oxygen Doping for Enhanced Catalytic Degradation of Organic Pollutants through Peroxymonosulfate Activation. *Environ. Sci. Technol.* **2018**, *52* (24), 14371-14380.

6. Li, X.; Ao, Z.; Liu, J.; Sun, H.; Rykov, A. I.; Wang, J., Topotactic transformation of metal-organic frameworks to graphene-encapsulated transition-metal nitrides as efficient Fenton-like catalysts. *ACS Nano* **2016**, *10*, 11532-11540.

7. Gao, Y.; Zhu, Y.; Li, T.; Chen, Z.; Jiang, Q.; Zhao, Z.; Liang, X.; Hu, C., Unraveling the High-Activity Origin of Single-Atom Iron Catalysts for Organic Pollutant Oxidation via Peroxymonosulfate Activation. *Environ. Sci. Technol.* **2021**, *55* (12), 8318-8328.

8. Luo, R.; Li, M.; Wang, C.; Zhang, M.; Nasir Khan, M. A.; Sun, X.; Shen, J.; Han, W.; Wang, L.; Li, J., Singlet oxygen-dominated non-radical oxidation process for efficient degradation of bisphenol A under high salinity condition. *Water Res.* **2019**, *148*, 416-424.

9. Wang, P.; Liu, X.; Qiu, W.; Wang, F.; Jiang, H.; Chen, M.; Zhang, W.; Ma, J., Catalytic degradation of micropollutant by peroxymonosulfate activation through Fe(III)/Fe(II) cycle confined in the nanoscale interlayer of Fe(III)-saturated montmorillonite. *Water Res.* **2020**, *182*, 116030.

10. Chen, F.; Liu, L. L.; Chen, J. J.; Li, W. W.; Chen, Y. P.; Zhang, Y. J.; Wu, J. H.; Mei, S. C.; Yang, Q.; Yu, H. Q., Efficient decontamination of organic pollutants under high salinity conditions by a nonradical peroxymonosulfate activation system. *Water Res.* **2021**, *191*,

116799.
11. Wu, L.; Li, B.; Li, Y.; Fan, X.; Zhang, F.; Zhang, G.; Xia, Q.; Peng, W., Preferential Growth of the Cobalt (200) Facet in Co@N–C for Enhanced Performance in a Fenton-like Reaction. *ACS Catalysis* **2021**, *11* (9), 5532-5543.
12. Wang, Y.; Liu, M.; Zhao, X.; Cao, D.; Guo, T.; Yang, B., Insights into heterogeneous catalysis of peroxymonosulfate activation by boron-doped ordered mesoporous carbon. *Carbon* **2018**, *135*, 238-247.
13. Wang, J.; Duan, X.; Dong, Q.; Meng, F.; Tan, X.; Liu, S.; Wang, S., Facile synthesis of N-doped 3D graphene aerogel and its excellent performance in catalytic degradation of antibiotic contaminants in water. *Carbon* **2019**, *144*, 781-790.
14. Song, H.; Guan, Z.; Chen, L.; Xu, H.; Xia, D.; Huang, M.; Li, D., Role of curvature in a carbon electronic structure under spatial confinement: Conversion of nonradicals to radicals. *Carbon* **2021**, *180*, 22-30.
15. Ma, W.; Wang, N.; Tong, T.; Zhang, L.; Lin, K.-Y. A.; Han, X.; Du, Y., Nitrogen, phosphorus, and sulfur tri-doped hollow carbon shells derived from ZIF-67@poly (cyclotriphosphazene-co-4, 4′-sulfonyldiphenol) as a robust catalyst of peroxymonosulfate activation for degradation of bisphenol A. *Carbon* **2018**, *137*, 291-303.
16. Chen, Y.; Zhang, G.; Liu, H.; Qu, J., Confining Free Radicals in Close Vicinity to Contaminants Enables Ultrafast Fenton-like Processes in the Interspacing of MoS_2 Membranes. *Angew. Chem. Int. Ed.* **2019**, *58*, 8134-8138.
